# The Corona-Eye: Exploring the risks of COVID-19 on fair assessments of impact for REF 2021

**DOI:** 10.1093/reseval/rvab033

**Published:** 2021-09-17

**Authors:** Gemma E Derrick, Julie Bayley

**Affiliations:** 1 Centre for Higher Education Research & Evaluation, Department of Educational Research, Lancaster University, LA1 4YD, United Kingdom; 2 Director of Research Impact Development, VC Office, University of Lincoln, United Kingdom

## Abstract

This paper assesses the risk of two COVID-19 related changes necessary for the expert-review of the REF2021’s Impact criterion: the move from F2F to virtual deliberation; and the changing research landscape caused by the COVID-19 crisis requiring an extension of deadlines, and accommodation of COVID-19 related mitigation. Peer review in its basic form requires expert debate, where dissenting opinions and non-verbal cues are absorbed into a groups deliberative practice and therefore inform outcomes. With a move to deliberations in virtual settings, the most likely current outcome for REF2021 evaluations, the extent that negotiation dynamics necessary in F2F evaluations are diminished and how this limits panelists’ ability to sensitively assess COVID-19 mitigation statements is questioned. This article explores the nature of, and associated capabilities to undertake, complex decision making in virtual settings around the Impact criterion as well the consequences of COVID-19 on normal Impact trajectories. It examines the risks these changes present for evaluation of the Impact criterion and provides recommendations to offset these risks to enhance discussion and safeguard the legitimacy of evaluation outcomes. This paper is also relevant for evaluation processes of academic criteria that require both a shift to virtual, and/or guidance of how to sensitively assess the effect of COVID-19 on narratives of individual, group or organisational performance.

## Introduction

As the global academic community works to recover and reorganize its research practice during and in anticipation of a ‘post’ COVID-19 normal, so too do existing evaluation and governance practices need to adjust. Although delayed, the UK’s Research Excellence Framework, and the evaluation of the Impact (capitalized to reflect the formal requirement within REF) will proceed, but with alterations to previously set, deadlines; census dates; evaluation practices; and how COVID-19 is taken into account as a reasonable adjustment and alteration to research productivity expectations pre-COVID-19. However, despite extended submission deadlines and census dates for Impact, and accommodation for changed Environments, there is little expressed understanding about how disruptions because of COVID-19 will be evaluated and how the adjustments necessary for panel evaluations to continue will influence this process. This paper considers the effect that changes necessary for a post-COVID-19-normal assessment processes may have on the evaluation of the non-academic, ex-post societal impact (the Impact criterion), as distinct from the two other REF components (Outputs and Environment). More specifically it will explore the connected implications of a) the shift from face-to-face to virtual review processes, and b) the challenges of judging the complex object of impact, now unsteadied further by COVID-19. It is important to explore both the theoretical and conceptual foundations and risk posed to the assessment of Impact by the COVID-19 crisis ahead of future empirical work exploring evaluative practice during the REF2021 evaluation process. The paper concludes with comments relating to potential risks associated with these aspects, and call for transparent expression of how these are to be integrated into the impact assessment in this unprecedented age. It further offers the cautions outlined in this paper to international counterparts embarking on national (or other such significant) impact assessments to enable fair, equitable and transparent processes to be established from the outset.

## Impact and formal assessment: a global enterprise

The deepening formality of impact evaluation has accelerated in the last two decades globally and is continuing to scale across nations with the UKs Research Excellence Framework arguably the most dominant expression to date. Germinated but paused by Australia in the early 2000s (and since reinvigorated), the principles of evaluating the non-academic benefits of university research are powerfully rooted on policy aims to demonstrate the contribution of academic research to broader society ((HEFCE) 2009). More specifically impact evaluation seeks to address 4 As - advocacy, analysis, accountability and allocation (Adam et al, 2018), more recently extended to include a further 2 A’s (acclaim and adaptation, https://www.rand.org/pubs/research_reports/RR3200.html) - offering a mechanism to not only inform government decision making about funding such as in the case of the UK REF, but also routinise expectations of ‘effect’ within the research sector. Notwithstanding broader debates about the drawbacks or merits of peer review compared to metrics-driven approaches (Wilsdon 2016), peer review remains a primary format of assessment, particularly in the UK. However, impact is far from a globally embedded or academically internalized aspect of research life, with considerable international variation in the extent to which an impact agenda exists/is implemented. Furthermore, impact evaluation is complicated by a variety of impact models, frameworks and heuristics available to understand it (Cruz Rivera et al. 2017). There is also criticism that REF is an expensive, over managerialist process that has little benefit for UK research (Watermeyer 2019). Accordingly whilst there is extensive coverage of how impact acts as a demonstration of 'public good' from research (Charities 2019) the operationalization of impact evaluation within academia continues to unsteadily walk the tightrope between social responsibility and instrumentalization.

**Impact assessment is still complex: the need for the Evaluator's Eye**

Despite the global implementation of Impact as a formalized evaluation criterion, Impact evaluation is still not a standardized endeavor and multiple models of impact assessment are used even within single domains such as health (Milat, Bauman, and Redman 2015; Greenhalgh et al. 2016; Cruz Rivera et al. 2017). As a result, the criteria and practice of consideration for such assessment is not fixed. Whilst the term 'impact evaluation' conceptually represents the calculable endpoint of research implementation, unlike traditional models of research excellence (such as outputs), there is no clear point at which impact is 'done'. Furthermore, positivist paradigms underpinning causation and measurability within impact assessment are not universally accepted across disciplines which are rooted in more transactional and subjective connections with society (Crossick 2016; Stern and Seifert 2016). Thus, research impact evaluation is a complex mix of primed expectations, disciplinary philosophy and multicomponent judgements of non-static objects.

Beyond models of Impact to understand its generation and aid its evaluation, Derrick (2018) examined how a focus on the practice of impact evaluation generates new models of impact appreciation that are specific to the place, and context of the evaluation. Here, the importance of panels developing an "Eye" or a strong panel identity and working methods was shown as crucial to reach a consensus to evaluate complex objects, such as non-academic, societal impact. Even though this research took place during the 2014 Research Excellence Framework, when Impact assessment was arguably more nascent, reviewers more novice and expectations less socialized, Impact definitions, exclusions and inclusions have remained substantively unchanged. Whilst some aspects have moderately evolved – such as improved accommodation of public engagement and impact on teaching in its REF2021 definition – the overall assessment objects within impact remain ambiguous, complex, resistant to simplistic mechanisms of academic evaluation and therefore in need of increased levels of deliberation within groups such as peer review panels. Within steady times, judgement of impact is difficult enough, but the extraordinary circumstances of

COVID-19 add challenges and complexities that are previously unexplored. It is therefore imperative that the risk that the new normal posed by the ongoing COVID-19 crisis to the evaluation of Impact be explored theoretically ahead of being tested empirically.

**COVID-19 and the UK Research Excellence Framework 2021**

COVID-19 has already had a significant effect on the immediate future of universities. Beyond the obvious (and significant) pressure on individuals to operate within a pandemic, there have also been numerous practical and staffing changes. For instance, universities have been routinely using their Polymerase chain reaction (PCR) machines and experienced staff and technicians to support the UK National Health Service (NHS) to deliver COVID testing. This reduces their capacity for non-COVID research, placing further strain on the future of UK university research. Expertise has also been redeployed towards the pandemic response, with such high-profile examples as vaccine development at the University of Oxford. Notwithstanding the implicit value of such resource diversion for public health, the cannibalisation of resources from non-COVID activities compounds the challenges for those already overstretched by, for instance, the time burden to convert teaching into virtual formats. Following a consultation about the REF-specific complications arising from COVID-19, Research England[1] made a series of alterations to the submission process to partially accommodate for these unprecedented pressures. These included a 4-month extension to the final submission date, and guidelines on issues such as inclusion of furloughed staff (point 18), delayed outputs (point 28) and changes in the research environment (point 64). For Impact (but not Outputs), a revised timescale - shifted back from 31st July to 31st December 2020 - effectively provided an additional 5 months for Impacts to develop, and with accommodation for accounts of disruption for resulting Impact Case Studies (ICS). In parallel, re-scheduled panel review processes have shifted to virtual deliberations. Whilst optimism pervades of a potential shift back to physical opportunities for debate, at time of writing, the persistency of COVID-19 means that online methods remain the primary and potentially only method for review.

**Why raise concerns?**

Such modifications, however practical, introduce altered accounts and new dynamics to the impact evaluation process. Group peer review[2] in its most basic form requires the presence of experts in a forum where debate is encouraged to achieve consensus and resolve dissent, with new approaches by necessity deviating from more traditional face-to-face (physical co-location) methods. Fundamental to fair decision making in impact assessment is recognition

[1] https://www.ref.ac.uk/media/1417/guidance-on-revisions-to-ref-2021-final.pdf

[2] For the purposes of this paper the terms: ‘groups’, ‘teams’ and ‘panels’, will be used to refer to the process of peer review conducted by more than one of expert through deliberation.

that the benefits of evaluative decision-making through peer review are not solely associated with the *nature* of the outcome (Derrick 2018), but also through demonstrations of *deep evaluative inquiry* via a deliberated and quality process. However, not only have review mechanisms needed to shift to virtual platforms, the subject of evaluation - Impact case studies - have themselves been affected by the COVID-19 crisis introducing need to account for changed trajectories and issues in evidencing. Given the financial and reputational weighting of the REF process, and the scale of the peer review machinery to reach judgements about ICS, it is essential that this process be transparent and attentive to the possibilities of bias, however unintentional. There is no criticism of the REF2021 for adapting in the face of COVID-19, but left unchecked these issues can too easily corrode the perceived legitimacy of – and academic trust in - evaluation of Impact. Accordingly it is the *absence of knowledge* of these aspects, rather than a presumption of deficient mechanisms, that underpins this paper.

To address this, we here draw attention to two separate (but connected) factors central to maintaining the legitimacy of evaluation in this COVID-adjusted context. Firstly, we explore the implications of (A) a move from face-to-face (F2F) to a virtual peer review evaluation, highlighting potential risks of presuming the benefits of physical review automatically transfer to online platforms; and (B) the challenges arising from the extended Impact deadline, both to the nature of impact case studies submitted, and the equitable judgement of impacts differentially affected by the pandemic. For each change we reflect on a series of considerations for the process of Impact peer review, followed by a set of recommendations.

**Change A: The move from F2F to a virtual peer review evaluation**

Panels faced with the evaluation of Impact do so amidst wider political pressure to ensure the ‘right’ outcomes are produced. Achievement of this goal is dependent not only on the content of the ICS, but also the combined understandings, shared experiences, existing collaborative relationships or acquaintances and resultant interactional expertise developed within groups at the time of the assessment (Derrick 2018). The shift to virtual platforms is eminently sensible in the current global context, and the increased efficiency combined with reduced cost makes the adoption of a virtual panel meetings an obvious choice during COVID-19, during which social distancing and restriction of travel prohibits F2F. However, whilst such a shift in normal times would be sufficiently paced to accommodate transitional planning, the ‘new normal’ has hastened the adoption of online infrastructures arguably without opportunity for due diligence on implications for academic governance and evaluation processes. Early indications show panellists reporting a decreased attention span (Zoom fatigue) during virtual peer review panel meetings (NIH Center for Scientific Review 2020; Singh Chawla 2021), and this move away from F2F evaluations further risk evaluations that are dependent on group interactions, such as REF2021.

**Consideration 1: Maintaining sufficient peer review deliberation in the virtual setting**

Within peer review decision making - where the decision is the collective responsibility of the group - the quality of the outcome is a direct consequence of the quality of the deliberation process between experts, or research peers (Lamont 2009; Derrick 2018). Communication, so central to this process, is in normal times facilitated by physical meetings in which individuals, now collected into a panel, debate and discuss the review object and reach a conclusion. The risk of *decreased* deliberation quality that is of particular concern for the evaluation of Impact which is, due to the increased range of experiences and expertise available to panels during the 2021 REF exercise, is already complex and difficult to achieve a group consensus and outcome. Thus, whilst a shift to virtual communication is undoubtedly the right course, caution is needed to avoid presuming vital deliberative processes are naturally transferred from physical to non-physical arenas.

Previous research on peer review decision making demonstrates an overall superiority of F2F methods. Studies show no significant difference in the quality of the decision making in teams using written (text-only) or audio-only communication (Gallo et al. 2013), but do show a benefit of adding video formats (including videoconferencing facilities) resulting in a significant improvement in the quality of team's deliberations and resultant strategic decision making (Baker 2002). Comparative research on the scoring patterns of peer reviews of traditional academic criteria has found only subtle differences in those between F2F and virtual panels; however, there was a decrease in discussion *quality* when deliberations were conducted virtually (Carpenter et al. 2015; Gallo, Carpenter, and Glisson 2013; Gallo et al. 2020). This is the case regardless of whether virtual deliberation is conducted using video conferencing, instant messages or other supported web technologies (such as parallel chat functions) (Carpenter et al. 2015; Gallo, Carpenter, and Glisson 2013; Gallo et al. 2020; Pier et al. 2017). Thus, regardless of the sophistication of web-technologies to attempt to re-create the benefits associated with F2F, F2F remains the preferred, and more efficient mechanism to support complex decision-making, with this effect amplified for new panel groups (O'Neill et al. 2016).

For peer review panels, F2F communication allows for better integration of expertise across panel members when evaluating as part of a group regardless of the group's history in working together, this integration of outside or minority voices is more difficult to encourage virtually. This is especially the case when non-verbal communication in virtual teams is minimized and yet still plays a large role in recruiting allies and including otherwise silent members during panel deliberations. More so than ever, the non-verbal, sometimes unconscious heuristics are essential for (large) evaluation panels to navigate the complex, and ambiguous object such as Impact, for assessment, and the availability of these cues are likely to be reduced when the deliberation is moved from F2F to a virtual setting.

Ensuring sufficient debate with all voices is a vital mechanism for avoiding groupthink (Derrick 2018), but this is made particularly challenging in virtual settings which denaturalize multiway discourse, limit the full airing of disagreements, and more readily default back to turn-taking monologues (Nemeth 1995; Nemeth and Rogers 1996; Yilmaz and Pena 2014). Interestingly, data suggests that differences in the quality of deliberations disappear for experienced reviewers[3] (Lam and Schaubroeck 2000; Schaubroeck and Yu 2017a), as long-standing teams are more resilient in virtual settings (Hollenbeck, Beersma, and Schouten 2012; Miles and Hollenbeck 2014), emphasising the importance of panel culture (Maruping and Agarwal 2004; Lamont 2009; Derrick 2018; Kozlowski and Bell 2013) in navigating complex evaluation objects. However, given the mix of new and experienced reviewers in REF2021, attention is needed on the effects of the virtual shift on the degree of panel cohesion ("temporal stability") where panel members are new to working together (Hollenbeck, Beersma, and Schouten 2012), and the extent to which heterogeneity in panel characteristics differentially affects their engagement with virtual methods (Schaubroeck and Yu 2017b).

Part of the practical moves towards more efficient evaluations comes from the explicit or implicit practice of decreasing the amount of deliberation though sidelining dissention or requests for clarification. Indeed, without the human distractions that are inevitable in F2F meetings, is a tendency towards streamlining discussions for the sake of it occurring online and therefore being too quick in discussions and not exploring things fully. In most situations, these additional needs for information are likely to originate from out-group members who are either impact novices with a low temporal stability and members of the interdisciplinary panel (who might create dissent by questioning the consensus otherwise established round disciplinary boundaries). More often than not, membership of these out-groups can perpetuate existing gender, racial or geographical biases in academia thereby limiting the ability for these individuals and groups to inform the evaluation process if there perspective counters the established consensus, and if there is a disproportionate focus on efficiency at the expense of complete, and fair deliberation. With the difficulties associated with considering non-verbal cues via virtual platforms (Lam and Schaubroeck 2000; Miles and Hollenbeck 2014), there is also less information available to the Panel Chair, or individual panelists to gauge when their actions or deliberations are indirectly or unfairly sidelining the opinions and inputs of out-group members, or when the virtual platform is being used to dissuade dissention or avoid conflict that could be used to improve the decision. Therefore, more care is required to ensure that the panel members are engaged with the evaluation process, their perspectives heard, and to detect non-verbal cues from

[3] Here 'experience' refers to previous experience assessing Impact; as well as experience working with other evaluators (resulting in a high degree of temporal stability) as part of the 2014 Research Excellence Framework.

panelists that indicate dissent from the consensus, but where panelists are dissuaded from raising dissent or needs for clarification verbally.

**Consideration 2: Low versus high temporal stability**

Temporal stability refers to the degree to which team members have a history of working together in the past and expect to be working together in the future (Schaubroeck and Yu 2017a; Hollenbeck, Beersma, and Schouten 2012). Teams that have a shared history of working together develop implicit norms and certain familiarities with one another, thereby reducing much of the uncertainty associated with how tasks are completed (Hackman and Katz 2010). Similarly, teams that expect to remain working together in the future are more motivated to invest the time to develop these norms to better facilitate how work is accomplished (Driskell et al 2003; Maruping and Agarwal 2004). Previous research has shown how teams with high degrees of temporal stability make better decisions than those with low temporal stability (Lu, Yuan, and McLeod 2012) and that complex decision making (Schaubroeck and Yu 2017b) including decision making around Impact, is made more difficult in less stable teams.

For REF2021, panels will be made up of both REF (and Impact) novices, as well as more experienced evaluators from REF2014, bringing challenges for the ‘temporal stability’ of panels. This is of course not uncommon within academia, with the frequent convening of new groups to assess applications, promotions and other such standard aspects of academic life. Impact and REF-novices have different deliberative and heuristic needs than evaluators who are either more experienced with the REF evaluation, or with working together. Accommodating these different needs, although minimal in F2F groups, is made more difficult if deliberations are conducted virtually (Driskell et al 2003; Schaubroeck and Yu 2017b). This is because panels with a high degree of temporal stability are able to bypass the benefits of F2F communication, since they are already familiar with the characteristics, expertise and potential contribution of each member, can anticipate more salient behavioural messages, the potential meanings of silence and therefore the reliance on the need for clarification (by confirming ‘have we decided on that?’) is low (Schaubroeck and Yu 2017b).

For the REF-panels, where panelists may already have existing academic relationships (e.g. collaborative, academic or professional) in addition to previous experience of REF-work, there is a need for a balance between; using the high temporal stability of in-groups to minimise the level of deliberation necessary to reach a decision, thereby increasing the ‘efficiency’ of the evaluation; and, promoting a longer, more 'inefficient’ deliberation capable for compensating for the low level of temporal stability between in- and out-groups but aiming to allow groups the time necessary to fairly and robustly assess the mitigation within each ICS. Whereas one option provides a quicker, more efficient outcome, it does so at the expense of developing the panel culture necessary to robustly evaluate Impact in a COVID-19 context. The other option may result in a 'better decision' due to the reduction in temporal stability through increased deliberation within the team but does so at the expense of time and efficiency.

**Consideration 3: Ensuring team cohesion and culture**

Teams that have a shared history of working together develop implicit norms and certain familiarities with one another, thereby reducing much of the uncertainty associated with how tasks are completed (Hackman and Katz 2010; Kozlowski and Bell 2013). This panel culture or 'Eye' (Derrick 2018) is an important driver of the evaluation, especially when tasked with complex decision-making. However, a low level of temporal stability (described above) in the first instance, does not imply that it will have an ongoing effect on the development of panel culture throughout the assessment process. Teams with less history of working together and who come together for the completion of a specific task are still able to work effectively together, as well as minimize the disadvantages associated with low temporality teams, but this is only possible if sufficient deliberation time is provided for the necessary development of the psychological linkages within teams including a willingness to contribute to a collective goal (Wiesenfeld, Raghuram, and Garud 2001). Increased and more mindfully managed deliberation, by the Panel Chair for example, would have the advantage of allowing groups to bring in voices and perspectives that would otherwise be sidelined because they originate from peripheral groups (low temporal stability) or that promote dissent in a consensus (judge) decision frame. This process, and the need to reach a consensus representative of all panelists, takes time that goes beyond a discussion that is driven solely by the need to produce an outcome.

**Consideration 4: Potential for conflicting decision frames in F2F versus virtual settings**

Previous research has shown how evaluation groups adopt a "solve" decision frame for aspects of non-academic impact (Derrick 2018) where the decision is framed around choosing the best option, rather than striving to reach a consensus as per a "judge" decision frame (Stasser and Stewart 1992). For the REF2021, the otherwise complex expert-led decision making around Impact is made more complex by the additional evaluation required around the mitigation needs within ICS. This increased complexity, requires more complex evaluation processes and tools to be applied by panel members. Further, whereas F2F teams are able to engage in more complex decision-making via a preferable 'solve' decision frame, to move to virtual deliberations complicates this process, as does the low level of temporal stability within panels. In a 'solve' decision frame, the team tries to find an optimal solution by seeking out information that reaches a demonstrably correct answer, thereby searching (and finding) information that allows the team to identify, and therefore defend, their choice as a decision (Weiss and Bucuvalas 1980; Stasser and Stewart 1992). Notwithstanding the implications of confirmation bias in group decision making

(Stasser and Titus 2003), there is clear need to understand the decision on, and processes arising from the adopted decision frame.

In addition, achieving a more ideal decision frame during virtual deliberations requires a greater level of complexity. A more time-consuming deliberation would be needed to compensate for the ease by which virtual platforms can silence non-peripheral, or even dissenting voices when the decision frame adopted by the group is seen to act against issues that are in need of more time, consideration and deliberation in order to reach an optimal solution. More likely, teams will tend towards a 'judge' frame which is based on the perception of a consensus and are more easily achieved for virtual settings. However, this goes counter to the solve frame normally achieved in F2F settings, which values processing all available information. Although it is debatable whether this differing decision frame would produce an alternative outcome, it does speak to the reliability of the evaluation process, the perception that the evaluation is fair and complete and therefore the outcomes accepted by the academic community. Therefore, even though solve frames are more harmful in virtual teams, they remain harmful so long as members do not work to compensate for the loss of social, non-verbal cues than support intensified information search and scrutiny (Dennis et al. 1996; Swaab et al. 2012) and therefore a desirable 'solve frame' which is more easily maintained in F2F settings. As such, where the considerations necessary to provide a robust decision are more complex, and where the deliberation is hampered by virtual communication, more work for panels, and panel chairs is required to encourage an effective and robust deliberation.

Of concern is research demonstrating that the medium of communication has an influence on this task of decision making (establishing the decision frame). In both virtual and F2F mediums, the decision frame adopted invokes different decision processes (Stasser and Stewart 1992; Stasser, Stewart, and Wittenbaum 1995; Stasser 1999). These differences are larger for more complex decision making, and when the team has uneven experience of working together between members (Stasser 1999). A shift to virtual raises particular query on key aspects of communication related to group's choice on decision frame: the speed in which a message is received (transmission velocity); the speed in which the receiver can obtain clarification of the meaning of a message (immediacy); and the extent to which multiple cues, verbal and non-verbal, are supported by the medium (symbol variety). The reduction in social cues available in virtual settings, compared to F2F, reduces the type of information search and scrutiny during decision-making necessary to support a 'solve' frame (Dennis et al. 1996; Swaab et al. 2012). Previous research has shown that F2F teams in solve frames make better decisions than virtual teams who adopt the same decision frame (O'Neill et al. 2016; Organ and O'Flaherty 2016). This is because with F2F settings, critical debate is welcomed as it allows for the exploration of dissent in building a dominant definition of the criterion through further deliberation (Nemeth 1995; Nemeth and Rogers 1996). In contrast, in communication mediums that are low on transmission velocity, immediacy of feedback and symbol variety – such as virtual mediums – reduced non-verbal social cues needed to interpret meaning result in more frequent requests for clarification from team members, as well as critiques of commonly held (group-based) or individual perspectives being seen as hostile. This can more readily lead to displays of defensiveness which interrupt the panel culture necessary for complex decision making (Derrick, 2018), and can interrupt the information seeking necessary to support a 'solve' frame as an effective approach to group decision making (Nemeth 1986). Thus, in virtual settings, critical discussion can unintentionally shutdown communications so needed to optimise outcomes and evaluation processes. In addition, the ability to manage conflict is also reduced in virtual teams who adopt solve frames. The question therefore remains as to how to create a virtual environment that balances the need for dissent, allows for the non-visual/verbal cues necessary to mediate misunderstandings stemming from dissent in a way that produces an optimal, and efficient outcome for the evaluation of the Impact criterion.

**Change B: Timescale extension for Impact**

To partially compensate for the disruption caused by COVID-19, the deadline for the Impact criterion was extended from July to December 2020. Notwithstanding the institution-level challenges of managing shifted deadlines, this change was a relatively straightforward adjustment to provide case study authors with more time to realise the Impact previously envisioned pre COVID-19, or at least have 'breathing room' to complete within the pressures of the pandemic. Within this however, timescale extensions have introduced two unanticipated dimensions of ICS; accountancy of 'affected case studies' (more commonly understood as mitigating circumstances) and the emergence of unanticipated COVID-led ICS.

This section discusses how the deadline extensions, and the absence of formal guidelines to panel of how to mitigate the COVID-19 effects on impact claims, compounds the existing complexity of decision-making to one where panels are asked to navigate the evaluation of Impact that is already ambiguous in concept, and now ambiguous in time.

**Consideration 5: Complexities introduced by mitigation**

For those existing ICS materially affected in some by COVID-19, institutions were given the opportunity to provide a 100 word 'affected case study statement' explaining how either Impact or evidence gathering were compromised. NB for the purposes of this article, from hereon we refer to the action of authoring the influence of COVID-19 as *mitigation*, and the subsequent panel evaluation of this as *mitigation judgement*. Within the context of ICS, mitigation can be best defined as the process by which the influence of COVID is reflected in accounts, more often than not expressing negative consequences to ICS. Guidance on both the extension and mitigation was clear that both were available options, to be used or not at the institution's discretion.

However, this procedural clarity belies the conceptual complexity of judging and narrating how cases are 'affected'. Revised REF2021 guidance expresses the nature and optional usage of extra time, the format of statements, but stops short of prescribing 'what counts' as legitimate mitigation. Instead, the requirement is for institutions to outline how the impact has been 'significantly affected by COVID-19' where such contextual information is required for panels to fully understand the case. As such, whilst extension is a clear-cut shift of date, the principle of 'affected' cases demands not only that institutions independently determine what counts as *significant*, but then successfully articulate these precise circumstantial detriments of COVID-19 blindly hopeful they have met the otherwise unscripted criteria for panel agreement. The incentives to maximise scores for, and indeed most successfully mitigate for disrupted cases, raises the very plausible chance that – in the absence of absolute guidance to the contrary - authors will have injected linguistic qualifiers into the ICS itself to express deviation from what 'would', 'could' or 'should' have occurred. Some accounts may conceivably now describe Impact as a past modal-verb (modal of lost opportunity, or 'what could have happened if only COVID-19 not occurred'), whilst others continue to account more traditionally and concretely about what has demonstrably changed.

Responsibility then shifts to panels who must add appraisals of extenuating factors to the already complex object of Impact. The process by which this will be achieved, or calibrated across cases and panels to ensure parity with unaffected cases, is yet unclear, bringing concerns over equitability and fairness in outcomes.

**Consideration 6: The potential risk of differential and divergent ICS Types**

In the absence of empirical evidence regarding the Impact submissions made by HEIs to the REF2021, the following section is presented in terms of 'risk' and 'potential for risk'. By bringing these potential risks to light prior to the evaluation process beginning (September 2021), the aim is to aid panels to evaluate submissions fairly, as well as offer HEI a framework with which to interpret outcomes. In addition, the section is offered as a conceptual framework for future studies seeking to analyse how COVID-19 mitigation was operationalized in REF2021 panels.

The extension of both the REF2021 census and submission deadlines, combined with the rapidly shifting UK research landscape in light of COVID-19 in 2020 (Watermeyer et al. 2021a); and the widely acknowledged competitively of UK HEIs (Watermeyer, 2019), means that it is reasonable to expect that HEIs would engage in changes to their ICS strategies in order to capitalize on their REF outcomes. A recent survey-based study of UK researchers showed an institutional de-valuing of research during the initial phases of the COVID-19 pandemic, except in relation to preparing REF2021 submissions (Watermeyer et al. 2021b). As for both individuals and organisations, altering research strategies in order to maximise outcomes from research performance-based audits is well documented (Watermeyer 2019). However, with the uncertainties to ICS development and completion, especially as it pertains to evidence collection, directly linked to the COVID-19 crisis, there is a risk that HEIs may use freedoms afforded by extended REF2021 census date and submission deadline to re-position final ICS selection and development in order to maxmise institutional performance. This potentially creates challenges for panels who, are already striving to operate as normal, in a new, virtual environment.

Whereas the extension of the impact window is a pragmatic accommodation aimed to aid submissions (HEIs and academics) in light of a complex and unprecedented circumstance, little guidance has been supplied to panels about how to mitigate the effects of COVID-19 within and between ICSs which have been differentially affected by COVID-19. Whilst in principle all cases should be judged on individual merit, this study envisages unanticipated challenges for panels to evaluate 3 divergent types of ICS emerging as a combined result of the COVID-19 pandemic and extended census/submission deadlines. We summarise the basic dimensions of these in Table 1, discussed further below. We recognise the extent to which ICS submissions to the REF2021 represent these divergent types will not be clear until all submitted ICS are published alongside of the REF2021 results[4], but consider the logical potential for their parallel existence sufficient necessitates attention on the potential evaluative risks faced by REF2021 panels.

Prior to the COVID-19 crisis, all case studies would have been characterised as Type 1, that is, unaffected by a global shutdown. However, COVID-19 potentially initiates two further types. Type 2 cases would be characterised as ‘continuing’, beginning before and running through COVID-19, requiring authors to react to changing (facilitating or inhibiting) circumstances. Type 3 cases in contrast would have *because of* COVID-19, primarily where research has been adopted into strategy, guidance or practice in support of public health. Sourcing corroboration for any Type of ICS is of course more challenging post COVID-19, particularly given the reduced capacity and availability of third-party testifiers, but Type 2 is arguably disproportionately affected by challenges of corroboration due to the changed circumstances *within* the ICS lifetime.

As impacts in Type 1 ICS occur before COVID-19, claimed effects would be untainted by the change in global circumstances, and thus evidence is (notwithstanding the difficulties obtaining evidence generally) a straightforward corroboration of ‘what happened’. The urgency and severity of the pandemic has led policy makers to draw on research with unprecedented expedience; uptake timelags so routinely expected in the translational cycle (Hanney et al. 2015; Morris, Wooding, and Grant 2011) have been vastly truncated for COVID-relevant research, resulting in the rapid acceleration of research usage with stronger effects than may have been originally envisaged. Accordingly, Type 3 ICS would have the advantage of accelerated effects with the prospect of far more real-time evidence. The

[4] A database of ICS submissions was established in light of the REF2014 exercise (https://impact.ref.ac.uk/casestudies/) and it is expected that the same will occur for the REF2021 submissions.

emergence of Type 3 is in line with acknowledged models of evidence-informed policy that reply on the sudden appearance of a social and economic need (Nutley and Webb 2000), pragmatic decisions shaped by political circumstance (Lomas 1997), or else streams converging (Kingdon 1984). Such effects could be found within any Unit of Assessment but could be most reasonably expected within health and medical related disciplines (eg. Main Panel A, UoAs 1-5), with key examples being the preponderance of public health modelling (e.g (Adam et al. 2018)) vaccine development, and initiatives around public behaviour and compliance.

However, Type 2 ICS would inherit a unique difficulty for REF2021 evaluation panels in comparison to the relative straightforwardness of Types 1 and 3. Revised REF guidance recognises that previously expected access or evidence may become unavailable due to COVID-19, and offers a mitigation mechanism for such cases. However, for those cases running through the pandemic, COVID-19 injects *corroboration uncertainty*, ie. The difficulty in judging if claimed effects post-COVID are substantiated by evidence collected pre-COVID, or proxy (or even absent) evidence, and/or the difficulty for evaluators to estimate the counterfactual against impact claims within ICS. Impact demonstrated pre-COVID-19 may continue along a planned trajectory (for example, continuing to increase), plateau (stall) or, depending on the nature of impact, conceivably be lost (ie. The planned opportunity, event, person or other activity on which the original impact trajectory was dependent was no longer available/possible, preventing the impact materialising in time for REF2021 submissions). For example, if an intervention was launched in the health service pre-COVID, with evidence showing patient benefit and service commitment to continued use, but healthcare staff become subsequently unavailable to comment due to the pandemic, to what extent could continued impact be presumed in the absence of further data? To what extent is evidence substantiating pre-COVID effects sufficiently corroborative of continuing or altered impact post-COVID? And in all cases where evidence is no longer available, how would less perfect 'proxy' measures or the overall absence of data be assessed? The existence of these possibilities demands new or alternative accounting, with associated mitigation judgment tools and adapted panellist behaviours to ensure fair and equitable assessment outcomes.

A notable consequence of the emergence of Type 3 is the opportunity for an institution to choose to submit a new ICS in place of one which has matured over a number of years (Type 1 or 2). This brings implications for staff engagement, recognition and buy-in to the broader research strategy. Ultimately the REF2021 process offers a route to financial security, and thus demands institutions optimize their submissions, this is therefore a competitive decision where COVID-specific case studies are perceived as having a greater potential to achieve a 4-star rating, by appealing to the "Corona-eyes" of panel evaluators (discussed below).

**Acknowledging individual experiences of COVID-19 - '*The same sea but different boats'***

A key related point for judging variations to ICS is that whilst COVID-19 has been a global phenomenon, effects on individuals vary wildly, with differential pressures by gender (Myers et al. 2020), employment sector, personal circumstances (such as caregiving), organisational decisions such as furloughing and many more. It is uncertain therefore how these heterogenous individual panel members experiences of COVID-19 may manifest in the review process, or if these will introduce personal assumptions into judgements of reasonable (versus disingenuous) accounts of disruption. A natural counterbalance to such biases is the development of a robust combined lens ("Corona-eye") of the group as a whole, able to more collectively assess impact claims and exercise fair evaluative caution in judging the epistemic legitimacy of claimed mitigation.

**The combined implications of a shift to virtual and changed Impact scope**

The judgement of ICS, and any associated mitigation in reference to COVID-19 is therefore dependent on a mixture of how cases (and mitigations) are articulated, the types of ICS submitted, how mitigation judgement is fostered and calibrated within panels, and how well institutional presumptions of 'what counts' match panel schemas. The joint potential for variation in authors approaches to narrating mitigation (Type 2), panel members' subjective judgments (*mitigation differential*) and unscripted accommodation of the three Types injects concern over parity of outcomes. The unprecedented global scale of COVID-19, and permeation into all aspects of life, risks elevating the perceived significance of work directly relating to the pandemic (Type 3) at the expense of comparatively downplaying unrelated research (Type 1 and/or 2).

Even with the presence of Impact evaluators from the previous REF2014, differences in evaluator experiences and definitions of 'excellence in impact', alongside different evaluation mechanics for REF 2021 (Derrick 2018) means there is a risk that group deliberation may continue to rely on the establishment of cohesive team behaviours to navigate the complexities of impact. Processes must now take account of conditional impact pathways and differentially affected Types of case, requiring the execution of different practices of mitigation. Whereas *normally*, F2F methods should offer the necessary conditions to address such complexity, there is little clarity on how virtual panels will be fully able to exercise the necessary agility in evaluative practice within the structures of virtual meetings.

**Aligning the panels: "Corona-eyes"**

The emergence of different impact Types, evaluated in altered fora, and with the high likelihood for incomplete substantiation or terminological inexactitude ('should have', 'would have') underscore the need to ensure panel processes are sensitive to, and transparent about the accommodation of, new complexities. Maximum mitigation is perhaps to be expected from institutions wanting to capitalize on an otherwise difficult

2020 by securing an equal (or greater) share of REF2021 rewards, this makes decision-making further complex through the need to judge the authenticity of such accounts. Indeed, appropriate, fair and harmonized evaluation of these issues requires panels to not only judge mitigation , but assess within ICS effectively, assessing the legitimacy of institutional claims about trajectory/disruption, whilst simultaneously separating screening out personal experience of the pandemic. We label this ability to judge impact according to pre-COVID best practice, whilst fairly and objectively accommodating the complexities arising from the pandemic and judging authenticity and proportionality of mitigation accounts as "*Corona-eyes*". We argue that panels cannot fully develop *Corona-eyes* without fully surfacing, and taking steps to address the challenges outlined here. It should be noted that whilst *Corona-eyes* is derived from considerations for REF2021, we believe it is applicable too to funding agencies and promotion panels in accommodating C.V. gaps over the forthcoming years, where the full effect of COVID-19 on individual careers and knowledge production are likely to manifest more profoundly.

## RECOMMENDATIONS

To partially remedy these potential imbalances, we summarise here a number of recommendations to support efficient and effective virtual evaluations, particularly in the assessment of complex objects (Impact). These recommendations are also summarised in Table 2.

### 1. Selecting an appropriate platform for panel deliberation

It is widely accepted that there is no, one technology that can support all stages of a decision-making process, nor replace all the benefits associated with F2F meetings. However, during the COVID-crisis, technology has adapted to the everyday needs of a larger-than-normal population, narrowing the differences between F2F and virtual decision-making. Whereas it is not the appropriate for this article to promote a particular platform over another, there are some characteristics of such platforms that will facilitate the evaluation of Impact using virtual mediums, while allowing panels the flexibility necessary to sensitively mitigate between different type of ICS resulting from the COVID-19 crisis. Where the F2F option is no longer available, agile and flexible teams that - when in coordination with a communication medium that is also agile and flexible - ensure efficient and high-quality decision-making in complex situations (O'Neill et al. 2016).

Any chosen platform therefore must enable sufficient flexibility of members to allow swift and clear communication, and emphasise resolution support. Platforms with asynchronous technologies or group decision support software (GDSS) best mimic F2F interactions, and commonly used approaches such as separation into smaller groups would also help guard against the isolation of any group members and management of large panels. Chat functions may also serve as a useful way to include more voices into these less naturalistic ways of communicating, and ensure technology does not reinforce unchecked dominant consensus. In addition, in line with the need to instil a desirable level of trust and engagement with the evaluation outcomes, the platform will also need to allow for a high level of security and confidentiality for evaluation deliberations, as well as the characteristics of the submissions.

**2. Additional calibration to accommodate mitigation and virtual decision-making**

The use of calibration exercises prior to the formal evaluation taking place within panels (Derrick and Samuel 2017), was used successfully during REF2014 as a mechanism to assist robust discussion around Impact, as well as provide an opportunity for panel members to clarify expectations and form a common lens to guide the impact evaluation (Derrick 2018). Calibration exercises, especially when the evaluation is anticipated as more complex, as was the case of Impact in REF2014, are used as an exercise in maintaining consistency and fairness in evaluation. REF2021 panels will of course undertake similar calibration exercises, particularly valuable for ensuring temporal stability and panel cohesion, but it is essential that additional calibration also attends to the combined challenges of virtual and complexified Impact. Here, it is vital that panellists be provided with sufficient detail and training to ensure deliberative practices are sensitive to the vulnerabilities of each type of ICS emerging as a result of COVID-19, as well as aware of how unconscious individual cognitive and emotional biases that may otherwise shadow how COVID-19 mitigation considered towards evaluation outcomes.

**3. Structured breakouts and/or discussion times**

It is unsurprising that one of the simplest strategies for enhancing deliberation is to enable discussion in within smaller groups. REF2021 panels are exceptionally large, while essential to ensure that the UoA has access the expertise necessary to evaluate all submitted Outputs, ICS and Environment statements, as well as the reap benefits associated with including international evaluators and non-academic experts, may prove burdensome when deliberations take place virtually. There are advantages, therefore associated with splitting the evaluation deliberations around Impact into smaller groups resulting in a micro-panel culture that is able to interact more through an online platform. Smaller groups are also able to more effectively shape their deliberation and decision-making heuristics around non-verbal clues that signal dissent, as well as be able to resolve dissent more openly than otherwise would be possible in a larger panel operating virtually. This indicates that smaller virtual sub-groups are able to exercise a larger dashboard of heuristics available to consider mitigation than for large virtual panels. Although it is not recommended that these smaller, sub-groups work autonomously from the larger panel, it presents a reasonable practice for the initial stages of the evaluation with, working groups then feeding outcomes and processes back to the main group at a later stage. Cross referencing of scores between these smaller groups, as well as utilising an ongoing form of calibration could also be used to ensure continuity of practice across smaller panels, UoA and Main Panels.

If utilising smaller sub-groups is not possible and a real-time deliberation desirable, using virtual platform in a similar way to how F2F operates risks isolating out-group members, and otherwise skewing the evaluation of Impact, and the consideration of COVID-19 mitigation towards otherwise dominant in-panel discourses. With this in mind, another recommendation is to host structured discussion times, (i) between panellists; (ii) between ICS under consideration; and (iii) around emerging mitigation characteristics underpinning the type of ICS emerging as a result of COVID-19. Whereas this technique would ensure that all voices are heard and to move towards the dissolution of out-groups to increase the level of temporal stability within the panel and move towards an inclusive consensus, it would also take a significantly greater amount of time that would contradict the desire for efficiency. In addition, managing a process that would involve all-inclusive deliberation through structured discussions, would also require a larger role for the Panel Chair (discussed below).

**4. Maximising the role of the Chair**

Managing group-peer review, from the perspective of the panel Chair who acts both as part of the panel's collective identity, but also mitigates the process from a top-down perspective, is an aspect of panel peer review operations that is often overlooked. In a post COVID-19 REF2021 the challenge for the Chair will to ensure the smooth virtual deliberation process but also to ensure that during these evaluations the move from F2F to virtual deliberations does not impede the ability of the panel to apply a responsible degree of mitigating judgement to the COVID-19 mitigation statements. This change places a greater responsibility on the Panel Chair as well as a greater level of Chair-awareness of the issues associated with evaluating Impact post-COVID-19.

Where the spontaneity of communication is enhanced in F2F, its absence, combined with the absence on non-vocal/visual cues within groups, restricts the efficiency of virtual teams. Ultimately it is the responsibility of the Chair to ensure the legitimacy of the panel's working methods, as well as the validity of its outcomes, and this presents extra challenges for panel Chairs when this process is undertaken virtually. The role of the chair also has a strong influence on interpersonal team dynamics and trust, which when combined with factors such as explicit management and an awareness of colleagues and their contexts; are essential factors in the efficient operation of virtual teams (Olson and Olson 2006). Managing deliberation over virtual settings can also be increasingly difficult for large, diverse teams, especially when effective leadership is highly dependent on quality interactions that are more difficult to establish, and to maintain virtually (Olson and Olson 2006). In virtual settings, it is also difficult to avoid hierarchical management styles that negate the chance for all voices to be heard, and regulating inter-dependencies between resources, task components and personnel. Indeed, centralized authority has a negative influence of team innovation, learning, adaptation and performance (Schaubroeck and Yu 2017a). The alternative, de-centralized authority, especially in the case of large teams (such as the REF2021 UoA Panels), is difficult to maintain and can further impede the temporal stability and construction of the mental modes necessary if this is conducted virtually. The challenge for the Chair, therefore, is to adopt a management style that promotes the inter/intra-personal team dynamics and levels of trust necessary to establish common ground, common conceptual frameworks ("Eye" (Derrick 2018)) within the time allowed, therefore ensuring an efficient panel deliberation process.

Whereas virtual evaluation panels may present the opportunity for a more efficient evaluation overall, minimising the effect of drawbacks associated with the move away from F2F deliberations requires a more dedicated management strategy within the panel. Administering this strategy, requires a larger than anticipated role for Panel Chairs in order to establish and maintain cohesion throughout the assessment process, and to enhance team interactions while ensuring that communication is fluid and not restrictive to any team members during the deliberative and scoring stages. Not only will the Panel Chair need to work harder, monitoring what is said, but who says it but they will also need to do so in an online environment that may prove challenging to monitor parallel lines of communication (the deliberative practice, alongside the chat-function), remain sensitive to non-verbal cues where visibility of the entire panel is limited, as well as to attribute deliberative points to individual panel members, and to silence overly dominant discourses. In light if this increased role, Panel Charis must be given the autonomy to adopt evaluation practices that go beyond the REF Panel guidelines put in place prior to the evaluation in place. In other words, a maximised role of Panel Chair must also come with the power to adapt evaluation processes in practice as difficulties emerge.

## DISCUSSION

This article has discussed the risks of a post- (or mid-) COVID-19 world to the peer review evaluation of the non-academic, societal impact (Impact) criterion under the UK's 2021 Research Excellence Framework. These risks reflect both factors that have underpinned the nature of the changing research landscape left over from an all but sudden halt to research and research impact activities as a result of the UK's COVID-19 lockdown in 2020; the altered rules and procedures for its evaluation as part of the UK REF2021; and the need to adopt alternative evaluation processes in a COVID-19 safe manner (i.e. the move from F2F to Virtual evaluations). We have sought to foreground the need to address the individual and combined effects of these issues, and have raised concerns about group decision making on a now even more complex object. More specifically, we wish to raise a dual call to ensure panels develop "Corona-eyes" to offset a range of potential risks to fair evaluation introduced by these new conditions, and to do so both ahead of data and alongside live monitoring of REF2021 evaluation processes to empirically examine these issues in practice.

The heterogeneity of Impact requires sector wide and multi-level impact literacy (Bayley and Phipps 2019a, 2019b), not only of the nature of research led change, but also the conceptual, operational and individual aspects which influence the curation and comparative appraisal of cases. Impact is a complex aspect of the research landscape, with formal judgement of it a chronologically more nascent skill than for parallel processes to judge academic outputs. Whilst impact shares many of the challenges of research assessment as a whole – for example the burden on institutions and the reductionism of metrics as proxies for 'Excellence' – it has unique characteristics which make it particularly vulnerable to the shift necessitated by COVID-19. The purpose of this paper was to underscore the interconnected risks facing panels undertaking impact evaluation in these circumstances, and provide insights necessary for appropriate pragmatic and governance modifications to maintain a panel's ability to fully exercise necessary reflexivity.

Most fundamentally, this article has highlighted the potential difficulties in adopting a blind *process-as-usual* approach to the peer review of the UK REF2021's Impact criterion. In addition, any hybrid approach (F2F and online) must further be assessed for any imbalances introduced by a mixed evaluative format, as well as the effects realized by the COVID-19 crisis. This article does not presume insouciance from REF or those undertaking similar evaluations, nor suggest that a shift to virtual is inherently negative. Indeed, virtual decision making processes are more inclusive of not only international voices, but those for whom physical attendance is more difficult or even prohibitive. Arguably therefore there is an advantage to the legitimacy and fairness of outcomes associated with a peer review process. Instead it presupposes that the pace of change in such unprecedented times may easily lead the implications outlined here to be overlooked, and the risk to the legitimacy of the evaluation outcomes, amplified.

In the absence of a global post pandemic reset, peer review is more likely to abandon the traditionality of the F2F structure and embrace the increased familiarity of a system facilitated partially or wholly online. The points raised in this paper are therefore applicable beyond the REF context, and offered as broader reflections for those transitioning to online evaluation processes, or needing to consider how to mitigate judgements of academic performance in light of the COVID-19 crisis. Whilst these concerns are primarily relevant to the UK REF process at this time, the seismic repercussions of COVID-19 mean that the implications for legitimate outcomes not only resonate across the research ecosystem now, but in future performance assessments.

## CONCLUSION

This is an unprecedented time, with unprecedented challenges across all areas of life. For impact assessment the challenges laid out here, and the associated suggestions for redress, offer an opportunity to establish a transparent, fair and robust process for evaluating impact and impact that '*should have been*'. Ultimately no approach is risk free, but it is in the remit of governing bodies to determine how these can be managed in practice, or more specifically develop "Corona-eyes" to offset them. Such practices are vital to retain community trust not only in the evaluative process, but in legitimation of the resulting financially and reputationally weighted outcomes

*Table 1: Three types of Impact Case Study arising from COVID-19*

| | **Description** |
|---|---|
| **Type 1** | ICS materially unaffected by COVID-19 (e.g. completed before the pandemic) |
| **Type 2** | ICS starting before, and *continuing through* the COVID period, straddling the pre- and during- COVID timescales. |
| **Type 3** | ICS emerging *as a result* of the new landscape, with impact arising because of COVID-19 (new or expediated). |

*Table 2: Summary of recommendations and relevance to combating risks to Impact*

| | **Recommendation** | | | | |
|---|---|---|---|---|---|
| | Appropriate platform | Re-calibration and training | Increased role for Chair | Structured discussion times | Greater autonomy for smaller sub-groups |
| Temporal stability | **x** | | | | |
| Development of shared values | **x** | **x** | | **x** | |
| Prevention of outgroup silencing | **x** | **x** | **x** | **x** | **x** |
| Preventing the use of proxies and shortcuts | | **x** | **x** | **x** | |
| Ability to assess COVID-effects | | | **x** | **x** | |
